# Performance of indirect immunofluorescence test and immunoblot tests in the evaluation of antinuclear and antimitochondrial antibodies

**DOI:** 10.55730/1300-0144.5512

**Published:** 2022-04-02

**Authors:** Ömer ÖZTÜRK, Selay DEMİRCİ DOS SANTOS DUARTE, Hatice YASEMİN BALABAN, Halis ŞİMŞEK, Burçin ŞENER

**Affiliations:** 1 Division of Gastroenterology, Department of Internal Medicine, Ankara City Hospital, University of Health Sciences, Ankara, Turkey; 2 Department of Medical Microbiology, Faculty of Medicine, Hacettepe University, Ankara, Turkey; 3 Division of Gastroenterology, Department of Internal Medicine, Faculty of Medicine, Hacettepe University, Ankara, Turkey

**Keywords:** Autoimmune hepatitis, primary biliary cholangitis, immunofluorescence test, immunoblot test

## Abstract

**Background/aim:**

Antinuclear antibodies (ANA) and antimitochondrial antibodies (AMA) have essential markers for the diagnosis of autoimmune hepatitis (AIH) and primary biliary cholangitis (PBC). These autoantibodies are detecting different laboratory methods. In this study, we studied the diagnostic performance of used methods in detecting ANA and AMA.

**Materials and methods:**

The autoantibody profiles of patients with AIH and PBC groups were analyzed with the indirect immunofluorescence test (IIF) and liver-specific antigens containing immunoblot test (IB).

**Results:**

There were 45 (87%) women in the study group and 8 (53%) women in the control group. The mean age of the patients was 50.5 ± 14.21 years old. The serum ALT and AST levels were higher in AIH, and ALP, GGT, and Ig M were higher in PBC. IIF test results among AIH/PBC groups; there was no difference in overall ANA positivity (p: 0.078). AMA was negative in all patients with AIH but positive in 83.3% of patients with PBC. IB test results among AIH/PBC groups; antibodies against PDGH, LKM-1, and Scl-70 were not observed in any patient with AIH/PBC. Except for M2 (p: 0.001) and M23E (p: 0.007) antibodies, there was no significant difference in antibodies between groups. Out of five PBC patients with negative AMA by IIF method, one was positive for AMA-M2, two were positive anti-gp210, and three were positive anti-M2-3E, but anti-sp100 was negative in all of them by the IB.

**Conclusions:**

AIH/PBC has complex associations with different autoantibodies, and some of these antibodies are not readily detected by the IIF test. IB assays with a wide variety of liver-specific antigens may be helpful in the diagnosis (especially in patients with AMA negative PBC) and follow-up in AIH/PBC patients.

## 1. Introduction

Autoimmune liver diseases (AILD) are a group of chronic inflammatory liver diseases, namely autoimmune hepatitis (AIH), primary biliary cholangitis (PBC), primary sclerosing cholangitis (PSC), and overlap syndrome (AIH associated with PBC or PSC) [1,2]. The etiology of AILD has not yet been described clearly; however, immunological mechanisms have been accused primarily in the pathogenesis of the disease. AILD can start at any age, and their diagnosis is based on clinical, radiological, histopathological, and serological investigations. Varieties of autoantibodies play significant roles in the diagnosis and classification of AILD. The level and presence of specific autoantibodies can also give information about the prognosis and response to the treatment. Therefore, autoantibody profiles in AILD are essential components of the diagnosis and follow-up of these diseases [2–7].

Indirect immunofluorescence assay (IIF) is the most commonly used screening technique to search for autoantibodies. Since there are problems in autoantibody detection and interpretation at routine serology laboratories, the usage of other methods, such as immunoblot tests (IB) together with IIF, may be helpful for accurate analysis of autoantibodies [8–10].

This study aimed to evaluate the diagnostic and prognostic value of IIF assay and IB test in AIH/PBC by focusing on the value of liver-specific autoantibodies measured by IB.

## 2. Material and methods

### 2.1. Study population

The ethics committee approved the study of Hacettepe University Hospitals, a tertiary-care hospital in Ankara, Turkey (study no: GO16/414). After the written informed consent was taken, 52 patients with the initial diagnosis of AIH/PBC/overlap syndrome (19 of AIH, 31 of PBC, and 2 of overlap syndrome) and 15 controls who have dyspepsia without other disease were included. AIH, PBC, and overlap syndrome were diagnosed according to the Paris criteria [11,12]. The liver biopsies were performed in all patients to confirm the diagnosis and make the differential diagnosis

### 2.2. Exclusion criteria

The exclusion criteria were malignancies, severe cardiopulmonary or renal failure, diabetes mellitus, rheumatologic diseases such as systemic lupus erythematosus, rheumatoid arthritis, scleroderma, or ankylosing spondylitis, decompensated cirrhosis, and concomitant other chronic liver diseases including nonalcoholic steatohepatitis, alcoholic hepatitis, hepatitis C virus infection (HCV), hepatitis B virus infection (HBV), Wilson’s disease, or hemochromatosis. Since the role of autoantibodies in the diagnosis of PSC is minor, patients with PSC were also excluded from this study.

### 2.3. Laboratory investigation

The serum samples were collected for serological tests and laboratory workup, including complete blood count, alanine aminotransferase (ALT), aspartate aminotransferase (AST), gamma-glutamyl transferase (GGT), alkaline phosphatase (ALP), albumin, total protein, total bilirubin, immunoglobulin (Ig)G, IgM serum levels and prothrombin time (INR).

The antinuclear antibody (ANA), antismooth-muscle antibody (ASMA), antiliver-kidney microsome type 1 antibody (LKM1), and antimitochondrial antibody (AMA) were analyzed by IIF microscopy (Euroimmun AG, Germany). The substrate combination consisting of human epithelial cells (HEp-2) and primate liver was used for the determination of ANA, and samples with serum titers ≥ 1:160 are considered positive. AMA, ASMA, and LKM were determined by using the basic mosaic profile containing HEp-2 cells, rat kidney-stomach-liver cells as substrates were according to the manufacturer’s recommendation. Both patients and controls were also screened by EUROLINE Profile Autoimmune Liver Diseases 14 Ag immunoblot strips (Euroimmun AG, Germany) the automated EURO Line Scan according to the manufacturer’s recommendation. This test kit contains strips coated with 14 different liver-specific antigens: AMA-M2, M2-3E, Sp100, PML, gp210, LKM-1, LC-1, SLA/LP, SS-A, Ro-52, Scl-70, CENP A, CENP B, and PDGH.

### 2.4. Statistical analysis

Statistical analyses were performed by using the statistical package SPSS 20.0. The statistical test applied was Fisher’s M-test for percentages, chi-square analysis for nonparametric variables, Mann–Whitney U-test for unpaired data, and nonparametric Wilcoxon’s-test for paired data. P-values <0.05 were considered statistically significant.

## 3. Results

There were 45 (87%) women in the study group and 8 (53%) women in the control group. The mean age of the patients was 50.5 ± 14.21 years old. As there were only two patients with overlap syndrome, they were evaluated separately. Nineteen patients with AIH and 31 patients with PBC were compared with each other. The baseline characteristics of the AIH/PBC patients are demonstrated in Table 1. The serum levels of bilirubin, albumin, IgG were similar among groups (p > 0.05), but ALT, AST was higher in AIH, and ALP, GGT, and Ig M were higher in PBC.

The distribution of each autoantibody by IIF among AIH/PBC groups is demonstrated in Table 2. There was no difference in overall ANA positivity (p: 0.078). AMA was negative in all patients with AIH but positive in 83.3% of patients with PBC. The ASMA was positive only in one patient with AIH, while LKM was negative in all study patients.

The distribution of each autoantibody by IB among AIH/PBC groups is demonstrated in Table 3. Antibodies against PDGH, LKM-1, and Scl-70 were not observed in any patient with AIH/PBC. Except for M2 (p: 0.001) and M23E (p: 0.007) antibodies, there was no significant difference in antibodies between groups. All patients with anti-SLA antibodies were also positive for anti-Ro-52 antibodies. There was no significant difference in anti-gp210 antibodies between groups, but all PBC patients with positive anti-gp210 antibodies had 5- or 6-fold increased liver enzymes and 2- or 3-fold increased IgM levels, and they were more resistant to treatment.

Out of five PBC patients with negative AMA by IIF method, one was positive for AMA-M2, two were positive anti-gp210, and three were positive anti-M2-3E, but anti-sp100 was negative in all of them by the IB.

When serum samples of the control group as analyzed by IB for 14 different liver-specific antigens, all of the antibodies were negative in all of them, except for 4 (26.6%) controls having M2-3E positivity.

According to the Paris criteria, two of our patients were compatible with overlap syndrome [11,12]. The characteristics of the first patient were as follows: A 39-year-old female had been referred to us due to elevated liver enzymes, ALT 288 U/l (5–40), AST 176 U/l (8–33), GGT 230 U/l (5–40), ALP 796 U/l (91–258) and bilirubin was normal, Serum IgG was 3420 mg/dL (700–1600), and IgM was 313 mg/dL (40–230). Other hepatitis etiologies were negative, and autoimmune serology showed ANA and AMA positive. Her liver biopsy showed some cholestatic and hepatic features together.

The characteristics of the second patient were as follows: A 58-year-old female patient presented to us with ALT 341 U/l (5–40), AST 150 U/l (8–33), GGT 1796 U/l (5–40), ALP 668 U/l (91–258) and bilirubin was normal. The serum IgG was 3640 mg/dL (700–1600), and IgM was 420 mg/dL (40–230). Other hepatitis etiologies were negative, and autoimmune serology showed ANA and AMA positive. Her liver biopsy showed some cholestatic and hepatic features together too. The distribution of each autoantibody by IB among two patients with overlap syndrome was PML which was positive only in the first patient, and Sp100 was positive in the second patient. Interestingly, all other antibodies were found negative in both.

## 4. Discussion

The serological detection of autoantibodies for AIH/PBC practice was done in clinical by various assays such as IIF test, IB assay, double-dimension immune-diffusion test (DID), counter-immune-electrophoresis (CİE), enzyme-linked immunosorbent assay (ELISA) (enzyme immune assay (EIA), line-immuno-assay (LIA) and radio-immune-precipitation assay (RIA) [1,2,7,11]. In this study, the performances of the IIF test and IB assay were evaluated for the diagnosis of AIH/PBC.

The IAHG guideline in 2004 concluded that the IIF test should be the first-line screening method in all patients who are considered to have AILD and so be investigated for ANA, AMA, SMA, and anti-LKM [13]. Consistently, the IIF test is the most commonly used screening technique for searching autoantibody [2,3]. IF detects full AMA as a pattern, and it detects AMA and ANA altogether. IIF is the standard gold method for the determination of ANA [13]. On the other hand, IF also has some limitations, such as labor-intensive technical steps and interpretation errors [12].

IB differs with respect to the number and type of included preselected mitochondrial antigens. While some include PDH-E2 as a sole antigen, the others include three or even more antigens among PDH E1-2-3, 2-oxoglutarate dehydrogenase (OGDC-E2), and branched-chain 2-oxo-acid dehydrogenase (BCOADC-E2). The main advantages of IB are that it is less operator-dependent and more automated. However, it also has limitations, such as factors related to commercial kits, the inclusion of a limited number of preselected antigen(s), or in a particular case presence of undefined antigens other than M2 [12–18].

AMA is an accurate diagnostic marker for PBC and can easily be detected by using the IIF method [15–18]. There are nine subtypes of AMA, but only four of them, namely anti-M2, anti-M4, anti-M8, and anti-M9, are associated with PBC [17]. The anti-M2-3E antibody is a subunit of the anti-M2 antibody. Despite the fact that IIF is considered a sensitive and specific test in the diagnosis of PBC, false positivity may occur due to other autoantibodies. Anti-M2 and anti-M2-3E antibodies are the most specific and sensitive autoantibodies for PBC [18–20]. In our study, AMA was positive in 83% of PBC patients by IIF test, while anti-M2 antibody and anti-M2-3E antibody positivity were significantly higher, and they were detected respectively at 58% and 87% by IB assay. However, anti-M2-3E antibody positivity can also be detected at a lower rate in healthy people, as shown in Pang et al.’s study [21] and the control group of 26.6% of our study.

AMA can be negative in PBC patients with clinical and histopathological findings of PBC [11,12]. In a study [20] including 127 PBC patients, AMA was negative in 36 patients by IIF test; however, eight patients were positive by enzyme immune assay (EIA), and 16 patients were anti-M2-3E antibody positive by IB assay. In our study, PBC patients with negative AMA by IIF method had positive 60% M2-3E antibodies by IB assay. Thus, we concluded that the determination of the anti-M2-3E antibody increases the diagnostic accuracy of AMA among PBC patients. Therefore, anti-M2-3E should be evaluated in patients with clinical suspension for PBC. However, more studies are needed on anti-M2-3E antibodies in AMA-negative PBC patients.

The dual presence of anti-Ro52 and anti-SLA was reported in patients with AIH [19]. Antibodies to Ro/SSA and anti-SLA are not cross-reactive to each other and are related with HLA DRB1_03. This dual presence of anti-SLA and anti-Ro/SSA may explain this shared HLA association [18]. Patients with dual antibody positivity had more severe PBC, more advanced histological features, and higher serum bilirubin levels than negative patients. These antibodies were also associated with relapse after corticosteroid withdrawal in AIH and PBC [18,19]. In our study, anti-Ro52 antibodies were positive approximately 30% of patients with AIH, 40% of patients with PBC. A study by Montana-Loza et al. [22], including 170 patients with AIH-type 1, revealed that anti-Ro-52 was present in 38.2% of patients, while anti-SLA was positive in only 15.8% in whom 15.2% of them had concomitant positivity for both antibodies. In another study, Saıto et al. [9] reported the concomitant presence of anti–SLA and anti-Ro-52 antibodies among patients with AILD. They also reported that the presence of anti-Ro-52 antibodies was found to be related to the severity of the disease, but in our study, we did not show such a relationship. As in other studies [18,19], we observed that all PBC patients with positive anti-SLA antibodies were also positive for anti-Ro-52 antibodies.

Anti-sp100 antibody can be a valuable serologic marker to confirm the diagnosis, especially in AMA-negative PBC patients with typical clinic or laboratory findings [23–24]. In our study, the anti-Sp 100 antibody was positive in 16% of PBC patients while negative in all AMA-negative PBC patients. Our study used a line immunoblot assay to detect liver-specific autoantibodies. However, the diagnostic method used in Mickiewicz P [25] and Manuel Lucena J [26] was ELISA. Thus, the differences might be attributed to different diagnostic methods used or to the different demographic characteristics of the patient groups.

Anti-gp210 is positive in approximately 25% of patients with PBC with up to 97% specificity [27,28]. Some studies showed that anti-gp210 antibodies in PBC patients indicated a poor prognosis with unfavorable disease course and rapid progression. Therefore, anti-gp210 antibodies are useful for monitoring treatment efficacy and early determination of patients who are at high risk for hepatic failure [29]. In our study, PBC patients with anti-gp210 antibody positivity had higher liver enzymes and a poor prognosis despite the treatment. Thus, anti-gp210 antibodies may have the potential to become an important prognostic marker in PBC patients [30–32]. Autoantibodies against 3-PHGDH were reported to be positive at 68.3% in AIH, 15.6% in PBC, 12.8% in chronic hepatitis C, 9.8% in chronic hepatitis B, and 2.5% in controls [10]. Yoshida et al. proposed that 3-PHGDH was not associated with the development of AILD [33]. We did not detect any autoantibodies against 3-PHGDH in healthy and AILD patients.

## 5. Conclusion

Numerous autoantibodies can be used for the diagnosis of patients with AIH/PBC. AIH/PBC has complex associations to different autoantibodies, and some of these antibodies are not readily detected by the IIF test. However, autoantibody titers and the detectability of antibodies can change according to the method and laboratories or even during diseases. IB assays with a wide variety of liver-specific antigens should be considered in the diagnostic portfolio of a laboratory, and these antibodies can be used to monitor the course of the disease and provide information about its prognosis.

**Highlights**

- Autoantibodies are useful for the diagnosis and classification of AIH/PBC.
- IIF assay is commonly used to search autoantibodies in the AIH/PBC, however, it may not detect some type of liver-specific autoantibodies properly, especially in patients with AMA negative PBC.
- IB test with liver-specific antigens can be used to detect these autoantibodies together with IIF assay, especially if the presence clinical suspicion is high.

## Figures and Tables

**Table 1:** The baseline characteristics of the patients.

	Study population (n: 50)	OİH (n: 19)	PBC (n: 31)	P
**Age (median)**	51 (21–73)	46 (21–62)	52 (25–73)	0.20
**Gender (M/F)**	5/45	2/17	3/28	0.92
**ALT (median/min-max)**	50 (8–864)	88 (8–864)	36(11–121)	**0.009** *
**AST(median/min-max)**	45 (12–1198)	64(12–1198)	35(18–102)	**0.03** *
**ALP (median/min-max)**	141 (56–991)	95(56–178)	188(96–991)	**< 0.001** *
**GGT (median/min-max)**	98.5 (11–570)	70(11–220)	135(11–570)	**0.01** *
**T. bil (median/min-max)**	0.75 (0.3–4.47)	0.73(0.3–2.81)	0.85(0.38–4.47)	0.55
**D. bil (median/min-max)**	0.2 (0.1–1.3)	0.3(0.1–1.3)	0.18(0.1–0.76)	0.86
**ALB (median/min-max)**	4.18 (2.5–4.9)	4.2(2.6–4.9)	4.17(2.5–4.7)	0.81
**Ig G (median/min-max)**	1580 (834–4700)	1660(836–4700)	1545(834–3300)	0.20
**Ig M (median/min-max)**	236 (58–1290)	172(58–268)	290.5(85–1290)	**0.001** *

* p < 0.05

**Table 2:** IIF assay results among AIH and PBC patients.

	Study population (n: 50)	AIH (n: 19)	PBC (n: 31)
**ANA (ANA unknown in three patients with AIH)**	**40/47(85.1%)**	15/16 (93.7%)	25/31 (80.6%)
**AMA (AMA unknown in five patients with AIH and 1 PBS)**	**25/44(56.8%)**	0/14	25/30 (83.3%)
**ASMA**	**1/50 (0.5%)**	1(5.5)	0
**LKM**	**0**	0	0

**Table 3:** Immunoblot assay results among AIH and PBC patients.

	Study population (n: 50)	AIH (%) (n: 19)	PBC (%) (n: 31)	Control (%) (n: 15)
**CB**	4	5.3	3.2	0
**CA**	2	0	3.2	0
**Scl-70**	0	0	0	0
**Ro52**	36	31.6	38.7	0
**SS-A**	22	26.3	19.4	0
**SLA/LP**	8	10.5	6.5	0
**LC-1**	8	10.5	6.5	0
**Gp210**	26	26.3	25.8	0
**PML**	14	5.3	19.4	0
**Sp100**	12	5.3	16.1	0
**M2**	40	10.5	**58.1** *	0
**M23E**	74	52.6	**87.1** *	26.6
**LKM-1**	0	0	0	0
**PDGH**	0	0	0	0

* p < 0.05
